# Gibberellic Acid Improves Photosynthetic Electron Transport and Stomatal Function in Crops That Are Adversely Affected by Salinity Exposure

**DOI:** 10.3390/plants14213388

**Published:** 2025-11-05

**Authors:** Jyoti Mani Tripathi, Bibi Rafeiza Khan, Rajarshi Gaur, Dinesh Yadav, Krishan K. Verma, Ramwant Gupta

**Affiliations:** 1 Biophysics and Photosynthesis Lab, Department of Botany, DDU Gorakhpur University, Gorakhpur 273009, India; jyotitripathi181@gmail.com; 2 Biology Department, University of Scranton, Scranton, PA 18510, USA; bibi.khan@scranton.edu; 3 Department of Biotechnology, DDU Gorakhpur University, Gorakhpur 273009, India; gaurrajarshi@hotmail.com (R.G.); dinesh.biotech@ddugu.ac.in (D.Y.); 4 Sugarcane Research Institute, Guangxi Academy of Agricultural Sciences, Nanning 530007, China

**Keywords:** gibberellic acid, photosynthetic leaf gas exchange, plant growth-development, DELLA, salinity

## Abstract

Soil salinity poses a critical threat to global agricultural productivity, exacerbating food security challenges in arid and semi-arid regions. This review synthesizes current knowledge on the physiological and biochemical impacts of salinity stress in plants, with a focus on the role of gibberellic acid (GA_3_) in mitigating these effects. Salinity disrupts ion homeostasis, induces osmotic stress, and generates reactive oxygen species (ROS), leading to reduced chlorophyll content, impaired photosynthesis, and stunted growth across all developmental stages, i.e., from seed germination to flowering. Excess sodium (Na^+^) and chloride (Cl^−^) accumulation disrupts nutrient uptake, destabilizes membranes, and inhibits enzymes critical for carbon fixation, such as Rubisco. GA_3_ emerges as a key regulator of salinity resilience, enhancing stress tolerance through various mechanisms like scavenging ROS, stabilizing photosynthetic machinery, modulating stomatal conductance, and promoting osmotic adjustment via osmolyte accumulation (e.g., proline). Plant hormone’s interaction with DELLA proteins and cross-talk with abscisic acid, ethylene, and calcium signaling pathways further fine-tune stress responses. However, gaps persist in understanding GA_3_-mediated floral induction under salinity and its precise role in restoring photosynthetic efficiency. While exogenous GA_3_ application improves growth parameters, its efficacy depends on the concentration- and species-dependent, with lower doses often proving beneficial and optimum doses potentially inhibitory. Field validation of lab-based findings is critical, given variations in soil chemistry and irrigation practices. Future research must integrate biotechnological tools (CRISPR, transcriptomics) to unravel GA_3_ signaling networks, optimize delivery methods, and develop climate-resilient crops. This review underscores the urgency of interdisciplinary approaches to harness GA_3_’s potential in sustainable salinity management, ensuring food security and safety in the rapidly salinizing world.

## 1. Introduction

Salinity stress is one of the major abiotic stresses that limit crop production worldwide [1]. High amounts of salts in the soil reduce water uptake, cause ionic toxicity, and activate production of reactive oxygen species (ROS) [2]. Salt stress disturbs physiological processes, such as photosynthesis, nutrient uptake, and stomata operation, which ultimately result in reduced plant growth, development and yield, and therefore compromise global food security [3]. Among the various environmental stressors, soil salinity has emerged as a critical ecological challenge, predominantly affecting tropical and subtropical regions. This phenomenon not only disrupts natural ecosystems but also significantly reduces agricultural productivity, thereby threatening food security, safety and economic stability [4,5].

Gibberellic acid (GA_3_) is a natural diterpenoid phytohormone. It is associated in the regulation of processes, such as seed germination, stem elongation, leaf expansion, flowering, and fruit development. It is also involved in stress responses in plants [6]. According to the United Nations World Population Prospects, the global population is estimated to reach 9.7 billion by 2050, peak at 10.3 billion in 2084, and slightly decline to 10.2 billion by 2100 [7]. Under salinity stress, GA_3_ activates protective mechanisms. It promotes cell division and growth, maintains tissue water content, and stimulates root and shoot growth, enabling plant adaptation to osmotic and ionic stress [8]. It also increases photosynthetic efficiency, synthesizes chlorophyll, stabilizes photosystem II (PSII) complex and electron transport rate in thylakoid membranes. GA_3_-induced defense is often associated with the degradation of DELLA proteins. These are negative regulators of plant growth that stimulate the expression of genes in stress response [9].

GA_3_ plays a pivotal role in various developmental processes, including seed germination, stem elongation, and flowering. Under saline conditions, GA has been shown to counteract growth inhibition by promoting cell elongation, enhancing nutrient uptake, and modulating stress-responsive gene expression [10]. The exogenous application of GA can mitigate oxidative damage by upregulating antioxidant enzymes, such as superoxide dismutase (SOD), catalase (CAT), and peroxidase (POD) [11]. In addition, GA_3_ regulates stomatal behavior by interacting with abscisic acid (ABA), ethylene (ETH), and calcium signaling pathways, so that gas exchange and water-use efficiency (WUE) can be maintained under saline stress condition [4,12]. By modulating biochemical and physiological pathways, GA_3_ enhances photosynthetic performance, controls energy expenditure, and improves the overall tolerance of plants in saline environments.

This review focused on the current knowledge of GA_3_-induced regulation of photosynthetic electron transport and stomatal function, highlighting underlying mechanisms, hormonal interactions, and potential applications for improving crop performance under saline conditions. One of the primary physiological effects of salinity stress is its interference with ion homeostasis [13]. The primary soluble salts responsible for soil salinity include sulfates, carbonates, chlorides, and nitrates, with key cations, such as potassium (K^+^), calcium (Ca^2+^), magnesium (Mg^2+^), and sodium (Na^+^) playing crucial roles in determining soil salinity levels [14]. Additionally, trace elements, such as selenium, lithium, boron, strontium, silica, fluorine, rubidium, manganese, aluminum, and molybdenum are often present in hypersaline soil, some of which pose significant toxicity risks to plants, animals, and humans [15].

The application of GA_3_ (likely a phytohormone or growth-alleviating compound) is noted as a potential mitigating intervention. The interplay of these factors culminates in severely reduced photosynthetic efficiency and plant growth under saline conditions [10,16]. This review aims to provide a comprehensive understanding of the physiological and biochemical consequences of soil salinity stress in plants, with a particular focus on the role of gibberellic acid (GA_3_) in mitigation of adverse effects. The discussion will explore the potential of GA_3_ as a regulatory molecule in stress adaptation, highlighting its capacity to enhance plant resilience efficiency under saline conditions for sustainable agriculture and socioeconomic development.

## 2. Salt Stress Effects

Regions suffering from water scarcity and inadequate drainage systems are particularly vulnerable to salinity stress, which further diminishes crop productivity [15]. Reports indicate that more than 6% of the world’s land area and nearly 19.5% of total irrigated land have already been negatively impacted by salinization, posing serious challenges for agricultural sustainability [17]. Salinity negatively impacts plant physiology by depleting chlorophyll content in leaves. This depletion occurs due to an increase in ethylene production, which subsequently inhibits chlorophyll biosynthesis [18]. Chlorophyll is an essential pigment involved in the light-harvesting process of photosynthesis, and its reduction leads to diminished photosynthetic efficiency, thereby impairing plant growth and productivity. The degradation of chlorophyll under saline conditions can be attributed to oxidative stress, which triggers the overproduction of ROS, leading to lipid peroxidation and chloroplast damage. Beyond its direct physiological effects, soil salinity induces profound modifications in plant metabolism, particularly in relation to photosynthesis.

Furthermore, high salt concentrations in the thylakoid membrane can lead to irreversible inhibition of photophosphorylation and electron transport, thereby disrupting the light-dependent reactions of photosynthesis [19]. This disruption significantly compromises ATP and NADPH production, which are essential for carbon fixation and overall plant metabolism [20]. Salinity-induced stress in crops is particularly detrimental when salt concentrations in the soil exceed the plant’s osmotic threshold. The osmotic stress caused by excessive salt concentrations leads to cellular dehydration, which disrupts turgor pressure and consequently limits cell expansion and division. Additionally, the accumulation of sodium ions (Na^+^) reduces plant growth and reproductive ability, thereby leading to significant downregulation in crop yield [10]. Enhanced uptake of Na^+^ alters the cellular ionic balance by reducing the concentration of calcium (Ca^2+^) and potassium (K^+^) ions [20].

**Figure 1:**
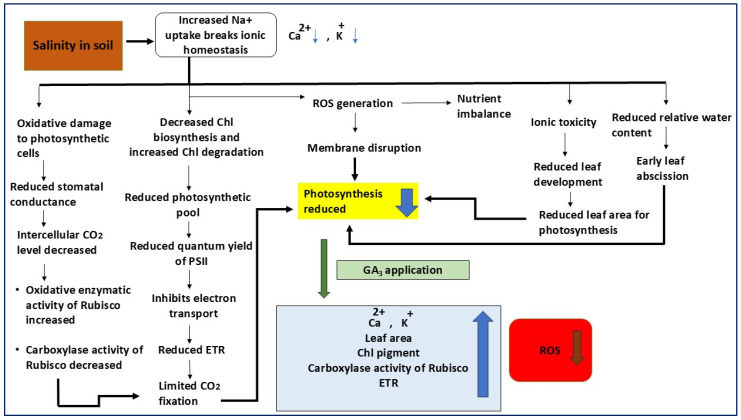
Proposed mechanism showing the effects of salinity stress and gibberellic acid (GA_3_) application on photosynthetic performance and ionic balance in plants. Under salinity stress, excessive Na^+^ uptake disrupts ionic homeostasis by decreasing Ca^2+^ and K^+^ levels, leading to oxidative damage, nutrient imbalance, and membrane disruption through enhanced ROS generation. These effects collectively reduce chlorophyll biosynthesis, increase chlorophyll degradation, and inhibit electron transport, resulting in lower PSII quantum yield, reduced carboxylase activity of Rubisco, and limited CO_2_ fixation. Salinity causes ionic toxicity, reduced leaf area, and relative water content, ultimately lowering photosynthetic efficiency. Exogenous application of GA_3_ mitigates these detrimental effects by improving ionic balance (Ca^2+^, K^+^), enhancing leaf area, chlorophyll content, and Rubisco carboxylase activity, as well as increasing electron transport rate (ETR). GA_3_ also reduces ROS accumulation, thereby restoring photosynthetic capacity and overall plant growth under salinity stress.

The activation of stress response mechanisms, including the synthesis of anti-desiccation proteins, plays a pivotal role in mitigating the physiological and biochemical disruptions caused by high salinity levels [21]. Salt stress often leads to severe osmotic imbalances, resulting in extensive physiological alterations, such as membrane destabilization, nutrient deficiencies, and impaired ROS detoxification. Additionally, it reduces photosynthetic rates, disrupts stomatal regulation, and alters antioxidative enzyme activities, all of which contribute to significant declines in plant health and productivity [22]. Calcium plays a crucial role in cell signaling and membrane stability, while potassium is essential for enzyme activation, stomatal regulation, and overall cellular homeostasis. The reduction in these ions under saline conditions disrupts numerous metabolic processes, leading to impaired physiological functions and reduced plant viability (Figure 1). Stress responses in plants are closely associated with polyamine accumulation, as observed in rice, lentils, and other crops [23]. Polyamines, including putrescine, spermidine, and spermine, act as signaling molecules that modulate antioxidant defense mechanisms and stabilize macromolecular structures, such as nucleic acids and proteins. An increase in polyamine synthesis under stress conditions enhances plant survival and adaptability [24].

Numerous studies have demonstrated that excessive salt stress leads to chlorophyll degradation and adversely affects electron transport due to surplus Na^+^ accumulation, thereby destabilizing the photosynthetic process. Elevated Na^+^ ion levels modify the K^+^: Na^+^ ratio, which in turn impacts energy-related processes in photosynthesis, including the degradation of photosynthetic pigments in cyanobacteria and higher plants. According to the study, salt stress at approximately 7–8 dS m^−1^ reduces carotenoid and chlorophyll levels in sugarcane (*Saccharum officinarum* L.) at different growth stages. Conversely, research on hot pepper (*Capsicum annuum* L.) has demonstrated a significant increase in chlorophyll and carotenoid content under 60 mM salt conditions [25,26]. This suggests that specific plant species or genotypes may possess inherent mechanisms that enable modulation of pigment biosynthesis and maintenance of photosynthetic efficiency under saline conditions. Consequently, carotenoid levels in plants subjected to salt stress could serve as valuable selection criteria for breeding salt-tolerant cultivars.

Moreover, gene-level salt tolerance has emerged as a promising strategy for developing stress-resilient crops. For example, in rice (*Oryza sativa* L.), the *OsSUV3* gene encodes a member of the *Ski2* family of DExH/D-box helicases, which enhances photosynthetic efficiency and strengthens antioxidant defenses under salt stress conditions [27,28]. Abiotic stresses lead to the activation of several enzymatic reactions, ultimately resulting in the reduction in photosynthetic efficiency [29]. These stress-induced alterations significantly impact the plant’s ability to assimilate carbon dioxide (CO_2_), regulate stomatal behavior, and maintain redox homeostasis, all of which are crucial for sustaining normal photosynthetic activity.

This reduction in photosynthetic activity contributes to estimated 50% decline in agricultural productivity, exacerbating concerns about sustainable food production [30]. Given that abiotic stresses account for significant economic losses in global agriculture, effective management strategies are critical to mitigating their adverse effects [31]. Increased soil salinity negatively affects key growth parameters, including plant length, leaf area-expansion, leaf number, and chlorophyll content. Several studies indicated that salinity stress alone accounts for 50% reduction in global crop production [32]. The most prominent effect is observed in stomatal conductance, which governs the opening and closing of stomata. Reduced stomatal conductance limits CO_2_ availability within plant cells due to the deactivation of key metabolic enzymes, such as Rubisco, sucrose–phosphate synthase (SPS), and nitrate reductase [33]. Rubisco, being the primary enzyme responsible for CO_2_ fixation in the Calvin cycle, is highly susceptible to stress-induced inhibition, leading to reduced carbon assimilation and impaired plant growth. The disruption of SPS and nitrate reductase activities further exacerbates the decline in photosynthetic efficiency by limiting carbohydrate metabolism and nitrogen assimilation, both of which are essential for plant energy balance.

During high saline stress, photosynthetic processes are severely impacted. The stroma enzymes generate thermostatic pressure, leading to further reduction in CO_2_ concentration within plant cells. High salt stress inhibits the enzymatic activity of Rubisco in in vitro and in vivo condition. The suppression of Rubisco under salinity stress results in lower rates of CO_2_ fixation, causing a shift in metabolic pathways toward alternative stress-mitigation mechanisms, such as photorespiration and the accumulation of osmoprotectants [34]. Conversely, an overall increase in Rubisco enzymatic activity could enhance the stability of plants suffering from abiotic stresses. In most C_3_ plants, leaf Rubisco content shows a positive correlation with phosphorus (P) and nitrogen (N) levels, except in glycophytic species, where this trend is reversed under salt stress conditions [35]. This contradictory relationship between Rubisco activity and N and P levels necessitates further exploration to elucidate its underlying mechanisms. Understanding these interactions could provide insights into breeding strategies for enhancing salt tolerance in crops. Under salt stress, two rice genotypes exhibit not only a negative impact on Rubisco activity but also a depletion of its substrate, ribulose-1,5-bisphosphate (RuBP) [36]. This indicates that salt stress impairs enzyme function and substrate availability, compounding the adverse effects on photosynthesis.

The combined effect of heat and drought stress amplifies the reduction in CO_2_ assimilation and places significant limitations on plant productivity. Different types of abiotic stresses have distinct effects on ferredoxin-NADP+ reductase (FNR) activity. In transgenic tobacco plants exposed to drought stress, FNR levels were significantly reduced. In contrast, under salt stress conditions, FNR activity increased in *Paeonia cathayana*, *Zea mays*, *Oryza sativa* and *Triticum* spp. This suggests that FNR may play a differential role in oxidative stress management depending on the type of abiotic stress encountered. Under osmotic stress in rice, the enzymatic activity of glyceraldehyde-3-phosphate dehydrogenase (GAPDH) was enhanced, while FNR levels were reduced [29]. This observation underscores the complex interplay between metabolic pathways under osmotic stress, highlighting the necessity for further research into how these stress-responsive enzymes contribute to plant adaptation and resilience.

## 3. GA_3_ Amelioration Mechanisms

**Figure 2:**
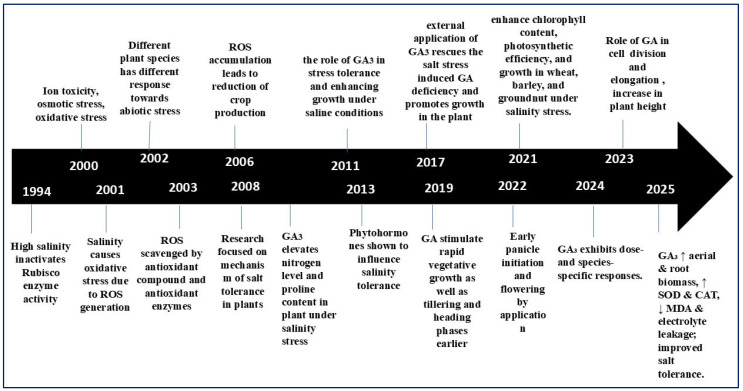
Timeline illustrating the progressive understanding of salinity stress and GA_3_-mediated responses in plants (1994–2025). Early studies (1994–2006) identified salinity-induced oxidative stress, ROS accumulation, and Rubisco inactivation as major constraints to plant growth. Between 2011 and 2019, research emphasized GA_3_’s role in enhancing nitrogen and proline content, regulating phytohormonal interactions, and promoting vegetative and reproductive growth under saline conditions. Recent findings (2021–2025) highlight GA_3_’s contribution to improving chlorophyll content, photosynthetic efficiency, antioxidant enzyme activity (SOD, CAT), and overall salt tolerance through species- and dose-specific mechanisms.

Plant growth regulators (PGRs) comprise a diverse group of molecules, including auxins, gibberellins, cytokinins, abscisic acid, and ethylene, each of which plays a distinct role in plant stress responses [37]. Among the plant growth hormones, gibberellins, particularly gibberellic acid (GA_3_), have long been known for their role in cell expansion, seed germination, and reproductive development. Recent studies have also revealed that GA_3_ plays a crucial role in regulating salt stress adaptation strategy (Figure 2). PGRs play a vital role in reducing the adverse effects of abiotic stresses, including salinity, drought, and temperature extremes [37]. Under stressful conditions, PGRs contribute to plant resilience by protecting against high temperatures, scavenging ROS, enhancing photosynthetic capacity, facilitating the accumulation of stress proteins, and regulating various metabolic functions [32].

The application of PGRs has emerged as a promising strategy for enhancing plant performance in saline environments. Recent studies have demonstrated that externally applied gibberellic acid (GA_3_) can mitigate the harmful effects of saline stress at various levels. Mechanistically, the effects of GA_3_ have been observed in the following ways, such as improving photosynthetic performance by maintaining the stability of PSII and protecting the pigment pool, enhancing carbon fixation capacity by increasing the amount or activity of Rubisco, and reducing ROS levels by activating antioxidant enzymes, such as SOD, CAT, and APX, thereby protecting the photosynthetic machinery. In a variety of crops, the use of GA_3_ as seed priming or foliar application has been reported to increase germination rate, seedling vigor, chlorophyll content, and photosynthetic rate. However, its effect depends on species, developmental stage, and depends on the dosage and application method [32,38]. Their influence extends beyond normal growth regulation, as they are also instrumental in improving plant tolerance to abiotic stresses, such as salinity [39]. Gibberellic acid (GA) exhibits growth-promoting properties in plants at different developmental stages. The mechanism of GA signaling operates through a tightly regulated balance between the repression of GA biosynthetic genes and the activation of catabolic enzymes and GA receptors, which modulate the bioactive form of GA within plant tissues (Figure 2).

A key component of this regulatory network is the DELLA family of proteins, which act as inhibitors of GA signaling. DELLA proteins suppress GA-induced growth and development, whereas gibberellic acid counteracts this inhibition by promoting the proteasomal degradation of DELLA proteins, thereby facilitating GA-mediated growth responses [40]. GA plays a crucial role in promoting vegetative and reproductive growth in plants by regulating essential physiological and developmental processes [41]. During seed germination, GA_3_ activates seed cells to synthesize mRNA, which encodes hydrolytic enzymes necessary for mobilizing stored nutrients. In saline conditions, GA_3_ enhances root and shoot elongation, increases leaf area, and improves photosynthesis rates by upregulating the enzymatic activity of invertase, which is involved in carbohydrate metabolism. These properties make GA_3_ an important regulator of plant resilience under salt stress conditions.

The GA_3_ receptor, GIBBERELLIN INSENSITIVE DWARF1 (GID1), is a soluble nuclear protein that interacts with the biologically active form of GA. The binding of GA to GID1 triggers a conformational change, leading to the formation of a GA-GID1 complex, which subsequently facilitates the recognition of DELLA proteins for targeted degradation. This interaction has been extensively studied in *Arabidopsis*, barley, tomato, wheat, and other plant species. Upon ligand–receptor binding, the GA-GID1-DELLA complex is recognized by the F-box component of the Skp1-Cullin-F-box (SCF) ubiquitin ligase complex, which mediates polyubiquitination and subsequent degradation of DELLA proteins via the 26S proteasomal pathway [41,42]. The degradation of DELLA proteins relieves their inhibitory effects on GA-responsive genes, promoting plant growth and developmental transitions. This intricate mechanism of GA signaling plays a fundamental role in multiple stages of plant growth and development, from embryogenesis and seed germination to root and shoot elongation, leaf expansion, stem elongation, floral induction, regulation of male and female flower numbers, seed development, trichome formation, and pollen maturation.

In particular, GA-mediated regulation of root architecture is crucial under saline conditions, as it enhances water and nutrient uptake, thereby improving plant survival in high-salinity environments. GA also enhances cell wall elasticity through the enzymatic activity of xyloglucan endotransglucosylase/hydrolase (XET) enzymes. Cell wall flexibility is a critical determinant of plant growth, particularly under stress conditions. In maize primary roots exposed to low water potential, a reduction in cell elongation and cell wall elasticity was associated with decreased XET enzymatic activity. Conversely, enhanced XET activity correlated with increased cell wall elasticity and cell expansion, underscoring the significance of GA in modulating cell wall remodeling [43]. In Pea plants, the effect of GA was studied under salinity stress condition in normal and saline soil, the GA pre-treatment increased the growth parameters in two pea varieties (Meteor-FSD and Samrina Zard), but the growth was more enhanced when GA_3_ was applied in combination with silicon (Si). The stomatal conductance (g_s_) in the leaves of both pea cultivars gets reduced under salt treatment, but the combined treatment of GA_3_ and Si enhanced stomatal conductance (g_s_). There was significant increase in tolerate variety (Samrina Zard) and a non-significant one in sensitive variety (Meteor-FSD) [44].

In *Stevia rebaudiana* Bertoni, 80 mM NaCl treatment significantly suppressed growth and photosynthetic efficiency, but 100 ppm GA_3_ treatment ameliorated these effects by increasing aerial biomass (54%), root biomass (31%), and leaf chlorophyll content also as compared with salty plants. Additionally, GA_3_ reduced malondialdehyde (MDA) (41%), hydrogen peroxide (34%), and electrolyte leakage (37%) [45].

In wheat, 150 mM NaCl reduced the growth and yield but moreover the wheat plant growth and yield were enhanced by the exogenous application of GA_3_ (0.75 mM), β-carotene (0.25 mM), GA_3_ + β-carotene (0.75 + 0.25 mM). GA_3_, β-carotene and their combination increased the antioxidant enzymatic activities of CAT, SOD, leaf ascorbic acid and total phenolics in wheat plants under salt stress conditions. Among the two varieties, Faisalabad-08 was more tolerant against salinity stress as whereas Galaxy-13 was least tolerant [8]. Moreover, foliar application of GA_3_ under saline conditions enhances proline accumulation in plants, which plays a crucial role in maintaining membrane integrity, improving permeability, and increasing the levels of essential macro- and micronutrients necessary for stress tolerance [46]. The accumulation of proline as a biochemical modification helps plants to maintain osmotic adjustment under salt stress conditions. Interestingly, studies on *Linum usitatissimum* suggest that GA_3_, in combination with calcium (Ca^2+^), significantly enhances proline accumulation, further improving stress tolerance [47].

In Maize, at moderate salinity level, GA_3_ application increased the activities of SOD, POD and CAT in the range of 12.23–38.12% relative to control plants. Under severe saline conditions, the maximum increase in SOD, POD and CAT enzyme activities was reported for GA_3_P + GA_3_FS, which were 73.03, 69.52 and 150.74% than respective control [16]. However, the role of GA in proline accumulation remains controversial, as studies in *Zea mays* and *Anabaena* indicated that GA treatment leads to a reduction in proline levels [48]. This contradiction suggests that the effect of GA_3_ on proline metabolism may be species-dependent and influenced by additional environmental factors. In wheat (*Triticum aestivum* L.) plants, low-dose of GA_3_ (10 ppm) combined with biochar improved plant water content (54.94–60.77%) and shoot fresh weight (34.47–43.18 g) under saline soil condition (EC = 5.11 dS m^−1^), while increasing chlorophyll and RWC [49].

In Turnip (*Brassica rapa* L.), salt stresses of 80 mM and 160 mM reduced the chlorophyll content, whereas the application of GA_3_ 2 mM was more effective and promoted the chlorophyll synthesis resulting in increased chlorophyll concentration as compared to control. The low dose of GA_3_ (1 mM) did not seem to be very effective [50]. In oats, under high salt treatment of 100 mM the growth parameters, such as seed germination (%), root and shoot length, tissue water content gets optimum reduced. But under the GA_3_ treatment of 100 and 150 ppm, the growth was increased. 100 ppm of GA_3_ concentration was not more effective compared to 150 ppm. NDO-2 variety of oats had better germination growth, vigor index and higher tissue water content when the seeds were treated with 150 ppm GA_3_ under different salinity conditions, but this amount of GA_3_ was not favorable for sensitive oats varieties because at high salinity level, 150 ppm GA_3_ reduced root growth [51].

For instance, in sugarcane (*Saccharum officinarum* L.), salinity has been shown to decrease carotenoid and chlorophyll content at various growth stages [52]. Seed germination is a critical initial phase in the plant life cycle and plays a pivotal role in determining the plant’s growth trajectory and successful establishment in the environment. Multiple factors influence plant development, but for seed germination, three fundamental environmental parameters are essential, such as light, moisture, and ambient air temperature. When these conditions are optimal, seed germination is facilitated through the activation of genes encoding ABA deactivation enzymes and the upregulation of gibberellin (GA) biosynthetic enzymes [53]. Despite the presence of these favorable conditions, high salinity levels impose significant stress on seeds, inhibiting germination and early seedling establishment. This is attributed to the suppression of GA biosynthesis, as reported in several studies. Under saline stress, seeds fail to germinate due to the inhibition of GA biosynthesis, particularly through the downregulation of the *GA3ox1* enzyme, which is crucial for converting precursors into bioactive GA. This suppression occurs at the transcriptional level, independent of ABA signaling, indicating a distinct regulatory mechanism governing salinity-induced dormancy.

In response to salinity stress, *Arabidopsis* exhibits increased expression of *NTL8*, a membrane-bound NAC transcription factor, during cold imbibition. This factor plays a critical role in seed germination under high-salinity conditions, independent of ABA signaling, suggesting a unique interaction between GA and transcriptional regulation [54]. *NTL8*-mediated germination control is directly linked to GA signaling, and its regulation has been observed in multiple plant species [31]. Additionally, RGL2, a DELLA protein, functions as a negative regulator of GA signaling and is essential in salinity–stress responses, where GA-mediated degradation of RGL2 is required for germination [9]. Emerging research suggests the existence of additional negative regulators of GA signaling beyond RGL2, highlighting an intricate network of GA-related stress responses. The application of exogenous GA_3_ has been shown to alleviate salinity stress in germinating rice seeds by compensating for reduced endogenous GA levels. This external GA supplementation effectively mitigates germination inhibition and promotes early seedling development under high-salinity conditions.

Gibberellins play a crucial role in reproductive development, particularly in regulating flowering time. Stamens serve as the primary site for bioactive GA synthesis, which is subsequently transported to other tissues to regulate male floral organ development and pedicel elongation. *GA20ox* and *GA3ox* are key enzymes involved in GA biosynthesis, and their regulation is influenced by adverse environmental factors [55]. Environmental fluctuations induce adaptive mechanisms in plants to optimize reproductive success under stress conditions. For instance, *Arabidopsis* responds to salinity stress by downregulating *FLOWERING LOCUS T* (*FT*) and *CONSTANS* (*CO*), transcription factors essential for floral transition. This suppression leads to delayed flowering and reduced reproductive success under saline conditions [56]. The inhibition of GA biosynthesis under salt stress contributes to increased sensitivity to salinity, primarily due to the accumulation of DELLA proteins, such as SLR1, which repress GA signaling and inhibit growth. In rice, *OsCYP71D8L* has been identified as a GA-inactivating protein that plays a crucial role in balancing growth and stress responses, thereby enhancing salt tolerance [57]. The reduction in GA signaling under salinity stress is strongly associated with enhanced salt tolerance in plants, suggesting that GA homeostasis is an adaptive mechanism regulating growth under adverse conditions.

In response to salinity stress, the bioactive GA concentration declines, leading to the inhibition of various growth-related parameters. This reduction in GA activity is likely due to a combination of decreased biosynthesis and increased catabolism. In several crops, including rice, the external application of GA has demonstrated significant benefits in improving salt stress tolerance. One of the primary mechanisms through which GA enhances stress resistance is by increasing chloroplast lipid production, which fortifies the plant’s structural integrity against salinity stress [58]. Salt stress negatively affects enzymatic activity and disrupts nutrient homeostasis in plants. However, in okra, foliar application of GA mitigates these adverse effects by restoring enzymatic balance and promoting growth under saline conditions [59]. Given its essential role in plant growth and development, GA is also involved in metabolic pathways that regulate cell division, seed germination, and overall physiological processes [60].

Under stress conditions, the inhibition of GA biosynthesis is a primary response, leading to reduced plant growth and the development of dwarf phenotypes. GA signaling suppression is linked to RNA processing modifications that are frequently observed during abiotic stress responses. This suppression is associated with the upregulation of *GA2ox*, *CsGA2ox8*, and *DELLA* genes, which encode GA-inactivating enzymes and inhibitors [61]. Gibberellic acid also plays a role in osmotic regulation under salt stress. In *Arabidopsis thaliana*, GA interacts with ethylene to regulate cellular expansion and division during salinity stress [62]. However, while the role of GA in growth regulation is well understood, its involvement in stress resistance pathways remains an area of limited exploration, necessitating further research [63]. Additionally, glycine betaine has been found to stabilize Rubisco structure under saline conditions, improving photosynthetic efficiency and stress resilience [64].

In response to environmental stress conditions, plants modulate bioactive GA levels and associated signaling pathways to optimize survival. One key adaptive strategy involves maintaining the dynamic source–sink relationship, wherein increased sucrose accumulation in source leaves leads to feedback inhibition of photosynthesis, further modulating stress responses [65]. Given the growing understanding of GA’s multifaceted role in plant stress adaptation, further investigations are required to unravel the complete regulatory network governing GA-mediated stress responses and its interactions with other hormonal pathways. Gibberellic acid priming has been shown to play a crucial role in mitigating the adverse effects of salinity stress on plant growth and development, particularly in cereal crops, such as wheat. One of the primary benefits of GA priming is its ability to positively regulate plant height under saline conditions. Salinity stress severely affects cell elongation and division, leading to stunted growth in many plant species. However, GA treatment has been found to counteract these effects by enhancing the expression of genes involved in cell wall loosening, thereby promoting stem elongation and overall plant height. In wheat, studies indicated that the GA concentration of 200 ppm is particularly effective in enhancing plant height in salt-intolerant cultivars of wheat. Additionally, higher concentrations of GA_3_ have been found to significantly increase the number of fertile tillers in wheat plants, particularly in salt-tolerant varieties, when compared to untreated controls [66].

In wheat, gibberellic acid (0.75 mM) and β-carotene (0.25 mM) concentrations were applied, along with their combined interaction by foliar treatments, the effects of GA_3_ and β-carotene foliar application, both alone and synergistically, on wheat plants subjected to salt stress (150 mM NaCl). Two wheat cultivars, Faisalabad-08 and Galaxy-13, were stressed with 150 mM NaCl in addition to control. Salt treatment increased the free proline level in both varieties; much rise was observed in Faisalabad-08 when treated with β-carotene. Highly significant difference was observed among both varieties for this attribute, as more for proline was recorded in Faisalabad-08. Foliar applications of β-carotene (0.25 mM), gibberellic acid (0.75 mM) and the combination of GA_3_ +β-carotene (0.75 + 0.25 mM) showed increase in free proline for both varieties [8].

GA_3_ priming has also been reported to improve shoot dry weight in salt-intolerant cultivars, suggesting its role in enhancing biomass accumulation under stressful conditions. This increase in shoot biomass is likely due to the ability of GA to enhance photosynthetic efficiency by upregulating chlorophyll synthesis and facilitating improved stomatal conductance, which collectively contribute to better carbon assimilation and growth under salinity stress. Furthermore, GA_3_ priming enhances the number of grains per ear under saline and non-saline conditions. Notably, it has been observed that GA priming is particularly effective in increasing grain weight in salt-intolerant wheat cultivars under saline conditions compared to salt-tolerant cultivars [67].

According to some studies, GA_3_ has also been found to be effective in improving transpiration rates under saline conditions. Water uptake and transpiration rates are critical physiological processes that determine plant resilience to environmental stresses. Under salinity, plants often experience osmotic stress, which restricts water uptake and leads to reduced transpiration. GA priming mitigates this effect by enhancing root hydraulic conductivity and improving stomatal regulation, thereby maintaining water balance and preventing excessive dehydration. In wheat, specifically, lower GA_3_ priming concentrations (100 mg/L) have been shown to improve transpiration rates in salt-intolerant wheat cultivars, whereas higher concentrations (150 ppm) have been more effective in enhancing transpiration in salt-tolerant cultivars [68].

Salinity stress is known to increase the sodium ion (Na^+^) concentration in plant shoots, leading to ion toxicity and osmotic imbalance. GA_3_ priming has been observed to reduce Na^+^ accumulation in plant tissues, thereby mitigating the detrimental effects of salt stress. This effect is achieved through the regulation of ion transporters, such as *SOS1* (*Salt Overly Sensitive 1*) and NHX (Na^+^/H^+^ antiporters), which facilitate sodium exclusion from the cytoplasm and maintain ionic homeostasis within the plant cells. The impact of salinity on tiller production has also been documented, with studies indicating that salt stress significantly reduces the number of tillers in plants. Primary and secondary tillers are particularly affected, often exhibiting inhibited growth due to salinity-induced hormonal imbalances. While GA_3_ priming has been shown to have a positive effect on tiller production, there is contrasting evidence regarding its efficacy when applied as a foliar spray. This suggests that the mode of GA_3_ application plays a crucial role in determining its effectiveness in promoting or inhibiting tiller formation [67].

### 3.1. Antioxidant System

Salinity stress induces oxidative damage in plants by increasing the production of ROS and promoting lipid peroxidation. However, the application of GA_3_ can mitigate these effects by enhancing antioxidant defense mechanisms [69]. GA_3_ treatment reduces ROS accumulation and lipid peroxidation levels, primarily through the upregulation of antioxidant enzymes, such as peroxidase (POX), polyphenol oxidase (PPO), superoxide dismutase (SOD), and catalase (CAT) [70]. The combined application of GA_3_ and salt stress induces plant signaling pathways that enhance antioxidant enzyme activity, reducing toxic compounds and ROS accumulation, thereby improving plant resilience under salinity conditions [46].

GA_3_ enhances nitrogen utilization efficiency (NUE) in plants under saline conditions by increasing nitrogen assimilation and improving nitrogen availability, which further promotes proline accumulation [54]. Additionally, GA_3_ plays a critical role in hypocotyl elongation by regulating calcium ion uptake. In *Stevia rebaudiana* Bertoni, 80 mM NaCl increased the ROS, whereas the GA_3_ (100 ppm) increased the antioxidant enzymatic activities, i.e., SOD (31%), CAT (11%) and PPO (24%), respectively [45]. In Indian mustard (*Brassica juncea* L.), the external application of salicylic acid (SA) and GA_3_ in combination increased the antioxidant enzymatic activities (PPO, POX, CAT, APX). Most enhanced effect was reported at 100 ppm GA_3_ and 0.4 µM SA as it increased the POX and APX activity. Moderate concentration of SA and GA_3_ can increase the POX activity at 8 dS/m and can also mitigate the salinity in India Mustard (Table 1) [6].

This inhibition further contributes to the reduction of photosynthetic efficiency under salt stress. Since ROS production is a byproduct of various metabolic pathways in mitochondria, chloroplasts, and peroxisomes, excessive ROS accumulation under stress conditions leads to widespread oxidative damage and reduced crop yields [71]. GA_3_ application under salt stress has been reported to alleviate these effects by improving stomatal function, reducing oxidative stress, and enhancing photosynthetic apparatus stability, thereby improving overall plant performance and productivity [72]. ↑, ↓ and → arrows indicated the increase, decrease and increasing values of the specific activities.

### 3.2. Photosynthetic Protection

**Figure 3:**
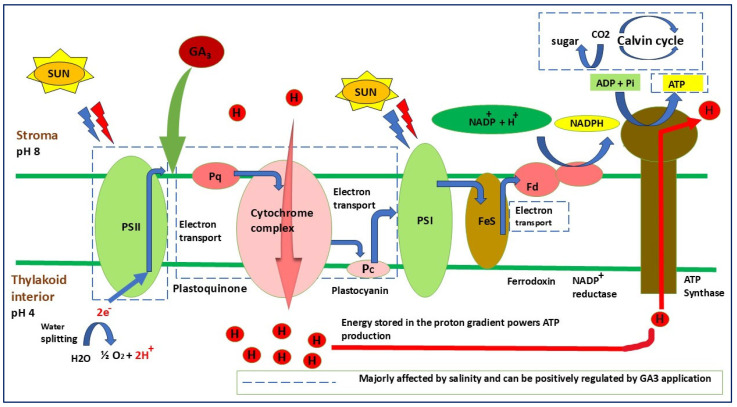
Schematic representation of the photosynthetic electron transport chain showing the primary sites affected by salinity stress and the modulatory role of GA_3_. Salinity disrupts electron flow between PSII, the cytochrome complex, and PSI, leading to impaired ATP and NADPH formation. GA_3_ indirectly enhances thylakoid membrane stability by promoting antioxidant protection, maintaining electron transport efficiency, and supporting the functional integrity of PSII and ATP synthase. The red lines indicate pathways majorly affected by salinity, while GA_3_-mediated recovery is highlighted to show its regulatory influence on photochemical energy conversion.

Salinity stress leads to an overall reduction in plant growth and development, resulting in significant reductions in crop productivity, particularly due to photosynthetic inhibition. One of the primary mechanisms through which salinity impairs photosynthesis by increasing sodium (Na^+^) accumulation in leaves, which disrupts the thylakoid membrane and leads to chlorophyll degradation [73]. This disruption results in a significant reduction in photosynthetic pigments. GA_3_ also reduces oxidative damage, indirectly protecting electron transport through thylakoid chain and PSII under stress conditions. PSII is the most sensitive site damaged under stress conditions; it has very important role during light reaction, mainly in electron transport processes (Figure 3). The photoreaction during light reaction requires coordination of the photosynthetic system in order to complete the normal linear electron transfer and also maintains the homogeneity for the fixation and reduction of CO_2_ in the dark reaction. In case of lettuce, exogenous application of GA_3_ led to an increase in WUE and A_N_/Ci ratio in the Grand Rapids cultivar as compared to other cultivars. Under saline stress, the application of GA_3_ resulted in higher values of Y_NPQ_ and Y_NO_, indicating that these cultivars possess greater protective capacity against the adverse effects of saline stress [8]. The primary reason behind this effect was due to an increase in regulated photochemical extinction quantum yield (Y_NPQ_), which dissipates excess energy as heat through the xanthophyll cycle [74].

The excessive uptake of Na^+^ ions alters the ionic homeostasis of Na^+^/K^+^ ratios, further reducing the bioenergetic processes of photosynthesis. This imbalance ultimately disrupts thylakoid membranes, leading to chlorophyll degradation in cyanobacteria and higher plants. Salinity-induced stress causes severe stomatal damage, degradation of chlorophyll content, and decline in other photosynthetic pigments [75]. These physiological changes result in reduced stomatal conductance, decreased CO_2_ fixation, and ultimately, lower crop yields. Additionally, salt stress primarily disrupts the functionality of PSII, reducing its efficiency by either oxidizing chlorophyll molecules or damaging chlorophyll-synthesizing enzymes [73]. However, GA_3_ has been shown to mitigate the negative effects of salinity on stomatal conductance and photosynthetic activity [10].

Under salinity stress, GA_3_ application increases stomatal conductance, enabling plants to maintain CO_2_ fixation and sustain photosynthesis even under high salt concentrations. Studies suggest that plants treated with GA_3_ under saline conditions exhibit higher chlorophyll content than untreated plants, indicating that GA_3_ has potential as a salinity-mitigating agent. Additionally, GA_3_ improves plastid ultrastructure morphogenesis, enhancing chlorophyll biosynthesis and overall photosynthetic efficiency under salt stress conditions. The pattern of ABA accumulation induced by salt stress did not change significantly after GA_3_ treatment, although GA_3_ could cause slight but consistent decrease in ABA concentration in the plant leaves. In GA_3_-treated plants, the generally lower level of ABA led to delayed activation of ABA-controlled stress responses, such as stomatal closure. This condition proved beneficial when there was no salt stress, as it allowed gas exchange and photosynthesis to continue. However, when salinity increased, it became detrimental, as rapid transpiration led to an increased accumulation of toxic ions in the shoot.

Several recent studies have revealed, such as in Maize, moderate salinity (6 dS m^−1^), and severe salinity (12 dS m^−1^) conditions, the exogenous application of gibberellic acid (GA_3_) by hydro- priming and foliar spray enhances the Chla, Chlb and total chlorophyll content by nearly 59, 140 and 78%, respectively, compared to control plants, which was decreased by 17, 30 and 20%, respectively, compared with no salinity treatment [16]. Photosystem II (PSII) is the primary component of the photosynthetic apparatus that experiences inhibition under environmental stress conditions, including salinity. PSII plays a crucial role in converting light energy into chemical energy, which is essential for maintaining photosynthetic efficiency [29]. Under high salt concentrations, several enzymes, including photosynthetic enzymes, become inhibited, which significantly impacts the efficiency of photosynthesis [29]. Salinity disrupts the overall growth and developmental processes of plants by inducing osmotic stress, which plays a vital role in maintaining water uptake and cell turgidity [76].

### 3.3. Stomatal Regulation

**Figure 4:**
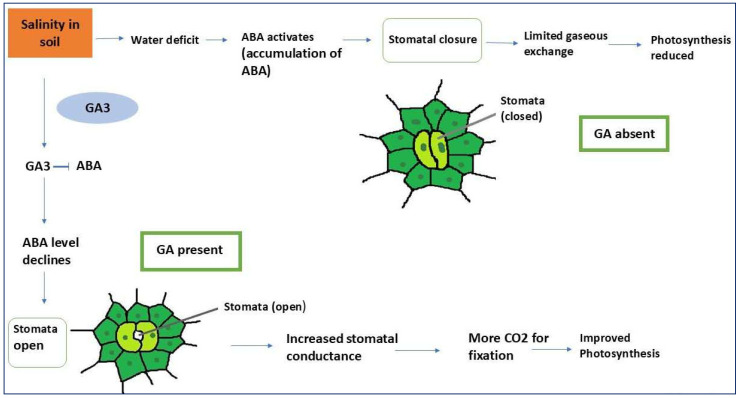
Interaction of abscisic acid and gibberellic acid in regulating stomatal dynamics and photosynthesis under salinity and water deficit stress. The figure depicts the antagonistic roles of ABA and GA_3_ in plant responses to soil salinity and water deficit. Under these stressors, ABA levels rise, triggering stomatal closure to limit water loss. This reduces gaseous exchange (CO_2_ uptake and O_2_ release), leading to diminished photosynthesis. Prolonged ABA accumulation exacerbates photosynthetic inhibition. Exogenous application of GA_3_ counteracts these effects by suppressing ABA levels, thereby promoting stomatal reopening. Open stomata enhance stomatal conductance, facilitating increased CO_2_ influx for carbon fixation and restoring photosynthetic efficiency. The GA_3_-ABA interplay highlights GA_3_’s role in mitigating stress-induced growth limitations. GA_3_ not only alleviates ABA-mediated stomatal closure but also improves overall plant resilience by balancing phytohormone signaling under adverse environmental condition.

The regulation of stomatal opening and closure is a complex process influenced by multiple environmental and endogenous factors, including hormonal signals. While light is a primary determinant of stomatal aperture, internal phytohormonal regulation plays a crucial role in modulating this response [77]. It is well established that ABA is a key mediator of stomatal closure, particularly under abiotic stress conditions, where its levels increase as part of the plant’s adaptive response. Salinity stress triggers significant rise in ABA concentration, leading to stomatal closure as a protective measure to minimize transpirational water loss (Figure 4). The ability of stomata to reopen after ABA-induced closure is dependent on the relative balance between ABA and other phytohormones, particularly auxins, such as indole-3-acetic acid (IAA), which can counteract ABA-induced stomatal closure at high concentrations [78]. This highlights the dynamic interplay between plant hormones in maintaining stomatal function under fluctuating environmental conditions.

Gibberellic acid has been implicated in promoting stomatal opening, primarily through its role in sugar accumulation within guard cells [79]. Guard-cell turgor is largely dependent on osmotic potential, which is influenced by the hormonal status of the cell. Under ABA dominance, the osmotic potential is maximized, leading to reduced turgor and stomatal closure [48,80]. In contrast, increased concentrations of GA and cytokinins reduce osmotic potential, thereby facilitating more rapid and sustained stomatal opening [81]. This indicates that GA may act antagonistically to ABA in regulating stomatal function. Furthermore, nitrogen availability plays a significant role in cytokinin biosynthesis, linking nutrient status to stomatal conductance (Figure 4) [82].

This ion imbalance leads to oxidative stress, resulting in decreased chlorophyll content and impaired stomatal responsiveness. Consequently, prolonged exposure to saline conditions can severely compromise plant growth and productivity. Recent studies indicated that the adverse effects of salinity stress on stomatal conductance can be mitigated through exogenous application of GA_3_ [83].

The growth-inhibitory effects of salinity have been observed at multiple developmental stages, with GA_3_ treatment shown to counteract these negative impacts effectively [42]. One of the primary mechanisms by which GA_3_ improves plant performance under salt stress through enhanced photosynthetic efficiency. By increasing chlorophyll content and maintaining chloroplast integrity, GA_3_ facilitates higher rates of carbon assimilation, which is essential for sustaining biomass production under stress conditions [40]. Additionally, GA_3_ promotes osmoregulation by increasing the levels of organic solutes, such as saccharides and proteins, leading to improved relative water content and expanded photosynthetic area. This suggests that GA_3_ applications can significantly enhance drought and salt tolerance by optimizing physiological and biochemical responses [40]. Moreover, GA_3_ has been reported to restore normal chlorophyll levels, promote vegetative growth, and improve physiological and chemical parameters in plants experiencing salinity stress. Its ability to regulate key metabolic processes, including nitrogen assimilation and carbohydrate metabolism, further underscores its potential as a critical modulator of plant resilience to saline environments. The interaction between GA_3_ and other phytohormones, particularly ABA, plays a pivotal role in determining the extent to which plants can adapt to osmotic and ionic stresses induced by salinity [42].

**Figure 5:**
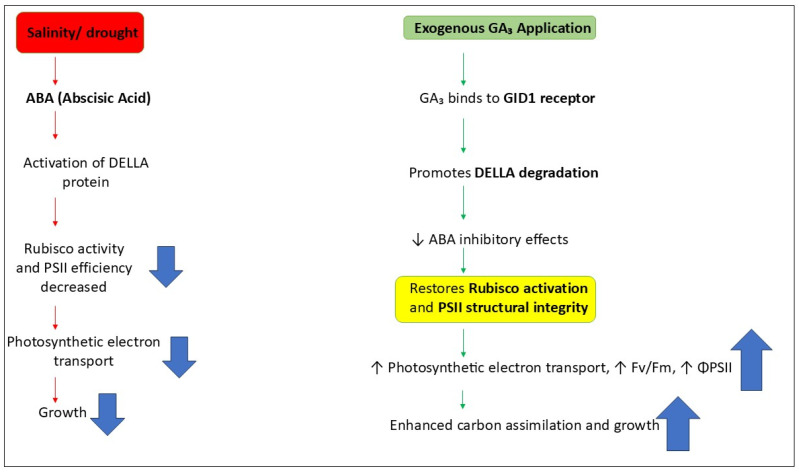
Diagram to demonstrate how GA_3_ restores photosynthetic function by regulating DELLA-ABA Rubisco. Red thin arrow shows the inhibition due to salinity stress, green thin arrow shows how GA_3_ degrades the DELLA and inhibits ABA inhibitory activity resulting in increased photosynthesis and promoting growth in plant under salt stress condition. Blue (up and down) arrows indicates the increase or decrease values of these activities.

The impact of salinity and drought stress on photosynthesis is largely mediated through reduced CO_2_ diffusion into chloroplasts. This reduction results from stomatal closure or limited stomatal aperture, which is governed by phytohormones, such as GA, and ABA (Figure 5) [84]. Additionally, salinity reduces mesophyll transport of CO_2_ and alters leaf photochemistry and carbon metabolism. These responses vary depending on the salt concentration, duration of exposure, and leaf age, with older leaves being more susceptible to salt-induced stress due to higher salt accumulation [85].

### 3.4. Hormonal Interactions

GA_3_ interacts complexly with other plant hormones, such as abscisic acid (ABA), auxin (IAA), cytokinin (CK), jasmonic acid (JA), salicylic acid (SA), ethylene (ET), and brassinosteroids (BRs) to regulate plant growth and stress responses under salinity stress. When plants are subjected to salinity stress, the ABA content in the plant increases, resulting in reduced GA synthesis and subsequent accumulation of DELLA proteins, the growth repressors of GAs, leading to inhibition of growth-related processes [86]. Exogenous applications of GA_3_ can be used to maintain the imbalance of GA_3_, reduce the content of DELLA proteins, activate GA signaling, and mitigate the inhibition of ABA on growth, which improves germination rate, photosynthesis, and plant vigor. Similarly, interaction of GA_3_ with auxin and cytokinins, which promotes the cell elongation, root and stem growth, which are generally suppressed under high salinity. Further, GA_3_ in conjunction with JA and SA, enhances SOD, CAT and APX activities and reduces ROS damage [87,88].

However, GA_3_ disrupts the ethylene signaling by inhibiting the premature leaf senescence caused by excess ethylene and retain the chlorophyll content and photosynthetic capacity [89]. Finally, the interaction of GA_3_ with brassinosteroids improves cell wall strength and photosynthetic potential because both hormones act via shared transcriptional regulator like BZR1 and DELLA [90]. Gibberellic acid interacts with DELLA proteins, abscisic acid (ABA), ethylene, and calcium (Ca^2+^) signaling pathways to regulate plant growth and tolerance to salt stress. In general, GA_3_ binds to its receptor GID1 inside the nucleus. Following this binding, the degradation of DELLA proteins begins, which normally inhibit plant growth. When plants are under salt stress, the amount of DELLA proteins increases. These proteins suppress the activity of genes related to photosynthesis, chlorophyll synthesis, and cell expansion. However, when GA_3_ is applied, it promotes the degradation of DELLAs and activates growth-related genes, such as Rubisco, chlorophyll-binding proteins, and expansins. In this way, GA_3_ restores the plant’s photosynthetic capacity and growth (Figure 5).

The GA_3_–DELLA system interacts antagonistically with ABA signaling [91]. During saline stress, the level of ABA increases, which activates *SnRK2* kinases and ABI5 proteins, thereby inhibiting plant growth. DELLA proteins further enhance this effect of ABA as they stabilize ABI5. However, when GA_3_ is applied, it reduces ABA synthesis by downregulating NCED genes and increases ABA degradation by upregulating *CYP707A* genes [92]. As a result, ABA levels decrease, relieving the growth inhibition, opening the leaf stomata, and improving water balance [93]. Similarly, GA_3_ also coordinates with ethylene signaling. Under saline stress, the increased activity of ACS and ACO enzymes leads to higher ethylene production, which promotes leaf senescence and chlorophyll degradation through the EIN3/EIL1 transcription factors [94].

DELLA proteins amplify this effect of ethylene because they stabilize EIN3, while the degradation of DELLA by GA_3_ destabilizes EIN3, reducing ethylene signaling, leaf senescence slow down and the stability of PSII is maintained. Furthermore, interactions and signaling between plant hormones play a crucial role in the adaptive responses of plants to adverse environmental conditions [38]. Hormonal crosstalk fine-tunes plant responses to stress by modulating gene expression, metabolic pathways, and physiological processes. For instance, under drought stress, ethylene production increases, which induces leaf senescence and disrupts the abscisic acid (ABA)-mediated regulation of photosynthesis during leaf development [95]. Ethylene–ABA interactions govern key aspects of stress physiology, including stomatal closure, antioxidant defense, and chlorophyll retention. The balance between these hormones ultimately determines the extent of stress-induced damage and the plant’s ability to recover from environmental stressors. This suggests that the relative concentrations of ethylene and ABA regulate plant stress responses under drought conditions [95,96].

Overall, GA_3_ establishes the balance between growth and stress tolerance by coordinating signaling pathways. The degradation of DELLA proteins (repressor/inhibitor of GA) suppresses stress-related signals of ABA and ethylene, while the Ca^2+^-dependent system stabilizes photosynthesis and antioxidant protection [97]. As a result, GA_3_ restores photosynthetic efficiency in plants, also maintains membrane integrity, and results in enhancement of overall physiological resilience under saline conditions. The interaction between GA and other hormones imply that GA_3_ is not only a plant growth-promoting hormone, but more importantly, a key hormonal integrator that controls the balance between growth and stress adaptation in plants.

### 3.5. GA_3_ Interactions with DELLA Proteins, ABA, Ethylene, and Calcium Signaling Pathways (Mechanistic Overview)

Salinity-induced plant responses regulated by GA_3_ are mainly dependent on tightly coordinated GA_3_-DELLA, ABA, ethylene, and Ca^2+^ signal transduction pathways. Under normal growth conditions, bioactive GA_3_ binding to the receptor GID1 forms a GA_3_–GID1 complex to degrade DELLA proteins, such as GAI, RGA, and RGL, through the SCF^SLY1/GID2 ubiquitin–proteasome pathway, thus releasing GA-responsive transcription factors, such as PIF3, PIF4, PIF5, and SPY, and then regulating the expression of downstream genes associated with cell elongation, photosynthesis, and metabolism. However, under salinity stress conditions, GA biosynthesis genes (*GA20ox* and *GA3ox*) are downregulated, and GA catabolism genes (*GA2ox*) are upregulated, resulting in the accumulation of DELLA proteins and growth inhibition [41,42].

GA_3_ application under salt stress reverts by promoting DELLA degradation and re-establishing normal PIF-regulated growth gene expression. The ABA pathway is antagonistic to GA by stabilizing DELLA proteins via SnRK2 kinases and ABI5, which repress GA biosynthesis and induce stress-responsive genes. The GA–ABA crosstalk operates hence as major regulatory switch between growth and stress tolerance [98]. Ethylene signaling intersects with the GA pathway via EIN3/EIL1 transcription factors, which coordinately with DELLAs control expression of leaf senescence and stress tolerance genes. GA_3_-induced DELLA degradation can repress EIN3 activity and hence prevent excessive ethylene-mediated growth inhibition and photosynthetic capacity loss [89].

Calcium (Ca^2+^) ion, which acts as a secondary messenger in plants and play important role in stress conditions. GA_3_ activates Ca^2+^ channels in the plasma membrane, increasing the Ca^2+^ level in the cytosol and enhancing GA signaling [99]. This Ca^2+^ activates Ca^2+^-dependent kinases, which increase the activity of antioxidant enzymes (SOD, CAT, APX) and help to maintain Na^+^/K^+^ balance via SOS pathway. Overall, GA_3_ coordinates all these signaling pathways to establish balance between growth and stress tolerance. The degradation of DELLA proteins which suppress stress signals associated with ABA and ethylene, while the Ca^2+^-dependent mechanism ensures stable photosynthesis and antioxidant protection [86].

Calcium signaling also converges hormone GA and ABA actions, where salinity stimulates transient cytosolic Ca^2+^ entry through channels, such as CNGC and TPC1, which activate Ca^2+^-dependent protein kinase (CDPK) and calmodulin-binding proteins (CBLs/CIPKs) [100]. These subsequently affect GA biosynthetic genes (*GA20ox*, *GA3ox*) and the stability of DELLAs through phosphorylation-dependent mechanisms. Ca^2+^ also interacts with ROS and ABA signaling and acts as a second messenger in ion homeostasis, stomatal regulation, and antioxidant defense [101]. Thus, GA_3_ signaling acts as a central integrator by connecting hormone pathways and calcium-related signaling to modulate the growth–defense trade-off under salinity. Mechanistic understanding of this network highlights key nodes, such as GID1–DELLA–PIF, ABA–SnRK2–ABI5, EIN3–DELLA, Ca^2+^–CDPK–GA2ox/GA3ox, which coordinate to regulate gene expression and physiological adaptation in salinity-exposed plants [100,102].

## 4. Conclusions

Salinity, a pervasive abiotic stressor, is a significant contributor to the global reduction in crop productivity. The excessive uptake of salt, particularly the accumulation of Na^+^, disrupts the normal ion balance within the plant, leading to decline in the uptake of essential ions, such as Ca^2+^ and K^+^. This imbalance exacerbates the detrimental effects of salinity on plant health, causing ionic, osmotic, and oxidative damage. These factors, collectively, contribute to decrease in seed germination rates, stunted growth, disrupted physiological processes, and ultimately, reduced crop yield. Salinity-induced ion imbalances and the resulting oxidative stress disrupt key physiological functions, making it critical factor in limiting agricultural output. This disruption impacts critical processes, such as nutrient uptake, water retention, and metabolic stability, further exacerbating the detrimental effects on plant growth, development and productivity.

Recent research findings clearly indicated that the GA_3_ plays a significant role in mitigating the harmful effects of salinity stress in various plant species. The application of GA_3_ under moderate (EC = 5.11 dS m^−1^) to severe (80 mM NaCl) salinity conditions resulted in remarkable improvements in plant growth, photosynthetic efficiency, and physiological stability. In *Stevia rebaudiana*, the use of 100 ppm GA_3_ increased aerial biomass (54%), and root biomass (31%), while chlorophyll content increased by approximately 40%. Additionally, it reduced oxidative stress, MDA decreased (41%), H_2_O_2_ (34%), and electrolyte leakage (37%), along with enhanced activities of antioxidant enzymes, such as SOD (+31%), CAT (+11%), and PPO (+24%). Similarly, in *Triticum aestivum* plants, application of low concentration of 10 ppm GA_3_ along with biochar improved plant water status and photosynthetic performance even under saline soil conditions (EC = 5.11 dS m^−1^), resulting in significant increases in RWC and fresh shoot biomass. Overall, these results indicated that GA_3_ exhibited a dose-dependent ameliorative effect in salt-stressed plants, mainly by maintaining osmotic, ionic balance, strengthening the antioxidant defense system and preserving the chlorophyll stability and WUE. Therefore, GA_3_ can be considered an effective bioregulator, offering a promising strategy to enhance salinity stress tolerance and yield potential of crops in saline soil regions.

Gibberellic acid, a plant growth hormone, has been shown to mitigate the toxic effects of salinity when applied externally, promoting plant growth and improving overall yield under such stress conditions. Specifically, the bioactive form of GA, GA_3_, enhances various physio-biochemical processes under salinity stress. GA_3_ functions as an antioxidant by scavenging ROS, promoting the degradation of DELLA proteins, and upregulating the expression of defensive genes. These genes are involved in synthesizing antioxidant enzymes and increasing the production of osmolytes, which play critical roles in osmotic regulation and stress mitigation. The application of GA_3_ has been found to play a dual role in salinity stress mitigation, not only by scavenging ROS but also by enhancing the synthesis of osmolytes, which contribute to maintaining cellular osmotic balance. GA_3_ mediated growth in the plant under salinity stress is dose-dependent. Low amounts of GA_3_ promote plant growth, photosynthetic capacity, and stress tolerance, whereas higher amounts can disrupt hormonal balance and ion homeostasis, leading to stunted growth or toxic effects it also interfere with auxin, ABA, and ETH signaling. Excess GA_3_ can lead to uncontrolled stem growth, chlorophyll deficiency, Na^+^/K^+^ imbalance, and oxidative stress, resulting in reduced plant growth and yield.

The beneficial effect of GA_3_ is limited to certain range (optimal dose range); beyond that, it can act as a growth inhibitor or be toxic. It is essential to identify this optimal concentration threshold for each crop species, as the response to GA_3_ depends on the crop’s genotype, developmental stage, and environmental conditions. This biphasic response suggests that the effect of GA_3_ greatly depends on its concentration, the crop species, and the intensity of salinity. To analyze the beneficial and inhibitory levels of GA_3_ in the plant under stress conditions, it is necessary to conduct systematic GA_3_ dose–response experiments across different types of crops, their developmental stages, and also at different salt concentrations. Such experiments provide physiological, biochemical, and molecular evidence for developing species-specific GA_3_ application strategies for different types of crops. They will be helpful in promoting the growth of plants under saline conditions. High salinity causes activation of several proteolytic enzymes, which degrades chlorophyll under saline conditions and reduces photosynthesis. When gibberellic acid was applied to the leaves of salt-stressed turnip, an increase in chlorophyll content was observed. This increase was maximal at the treatment concentration of 2 mM GA_3_ [50].

Under stress conditions, proline accumulation increases as stress response but the role of GA_3_ in regulating proline accumulation under stress conditions is not yet clear. While some studies indicated that the GA_3_ can enhance stress tolerance through osmolyte modulation, certain evidence from *Zea maize* and *Anabaena* suggests that GA_3_ treatment may decrease proline levels. Further research are needed in this direction to understand the mechanisms of proline regulation under abiotic stress conditions and to determine how GA_3_ affects proline regulation in various plant systems during stress conditions. GA_3_ application is highly dose specific and also depends on species to species. Low dose of GA_3_ could promote the growth but high dose cause the growth inhibition. In Oats, high dose of GA_3_ (150 ppm) reduces the plant growth especially root length was reduced. Root growth is the most important parameter for the survival of any plant.

In tomato, the pattern of ABA accumulation induced by salt stress did not change significantly after GA_3_ treatment, although GA_3_ caused slightly but consistent decrease in ABA concentration in the leaves. In GA_3_-treated plants, the generally lower level of ABA led to delayed activation of ABA-controlled stress responses, such as stomatal closure. This condition proved beneficial when there was no salt stress, as it allowed gas exchange and photosynthesis to continue. However, when salinity increased, it became detrimental, as rapid transpiration led to an increased accumulation of toxic ions in the shoot.

In Pea plants, the photosynthetic activity gets reduced by 66% in leaves of solely salt-treated plant as compared to control. When NaCl and GA_3_ together applied, it enhanced the photosynthetic activity compared to plants treated only with salt. However, it remained lower than control or solely GA_3_ treated plants. This indicates that GA_3_ plays a regulatory role in improving the net CO_2_ assimilation of pea plants under saline conditions. By influencing the expression of defense-related genes, GA_3_ fortifies plants against salinity and drought stresses, highlighting its potential as a valuable tool in improving stress resilience in crops. Salinity adversely impacts the overall growth and development of plants, primarily due to the decrease in water potential and the increased uptake of salt, which leads to the reduction in the RWC of plant cells. This effect is compounded by the breakdown of thylakoid membranes, which impairs photosynthetic efficiency. As a result, critical photosynthetic parameters, such as chlorophyll content, are diminished, and leaf area is reduced. However, specific mechanisms, potentially modulated by GA_3_, help plants to mitigate salt-induced stress. These mechanisms include an increase in stomatal density, enhanced membrane permeability, and an upregulation of antioxidant enzymatic activity. Additionally, the accumulation of proline, a key osmolyte, is enhanced by GA_3_ application, aiding in osmotic regulation and protecting the plant from further stress.

In Maize, hydropriming (100 ppm GA_3_) and water foliar spray (100 ppm) can be applied but application of GA_3_ by foliar spray under moderate and severe salinity is found to be more effective than hydropriming [16]. These findings suggest that the improvement in various physiological and biochemical parameters, such as stomatal density, antioxidant activity, and proline content, can significantly contribute to the plant’s ability to tolerate salinity stress.

Therefore, it can be concluded that the response mechanisms of plants to salinity stress vary across species, and their ability to stabilize photosynthetic electron transport and maintain metabolic homeostasis is influenced by the specific abiotic stress conditions. These species-specific responses highlight the complexity of plant stress tolerance mechanisms, necessitating further investigation into the molecular and physiological processes that enable plants to adapt to and thrive under challenging environmental conditions. The differential responses of various species to salinity stress suggest that tailoring stress management strategies based on species-specific mechanisms will be essential for optimizing crop productivity under saline conditions. Genetic engineering approaches aimed at overexpressing such stress-responsive genes could offer new avenues for enhancing crop resilience to salinity. By leveraging advancements in molecular biology and genomics, researchers can identify key regulatory networks involved in salinity adaptation, thus paving the way for the development of genetically improved crop varieties.

## 5. Future Prospective

The escalating threat of soil salinity to global food security demands innovative, multidisciplinary strategies to enhance crop resilience. Building on the insights from this review, several future research directions emerge, integrating the work within this review and novel avenues for exploration.

### 5.1. Genetic and Biotechnological Innovations

The identification of salt-responsive genes (e.g., *OsSUV3*, *OsCYP71D8L*) and their regulatory networks opens opportunities for precision breeding. CRISPR/Cas9-mediated genome editing could target DELLA proteins or GA biosynthetic pathways (*GA20ox*, *GA3ox*) to engineer salinity-tolerant crops. Cellular and tissue-level mapping of ionic toxicity (e.g., Na^+^, Cl^−^) will clarify spatial stress responses and set targets for tolerance improvement. Additionally, transcriptomic and proteomic studies could unravel species-specific GA signaling cascades, enabling tailored genetic modifications. For instance, overexpressing GA receptors like *GID1* or silencing GA catabolic enzymes (*GA2ox*) may amplify GA-mediated stress resilience. Biotechnological tools, including gene-editing and high-throughput screening, could identify novel negative regulators of GA beyond DELLA proteins, refining our understanding of growth–stress trade-offs.

### 5.2. Elucidating Molecular Mechanisms of Hormonal Cross-Talk

The antagonistic interplay between GA and ABA in regulating stomatal conductance and stress adaptation warrants deeper investigation. A critical gap remains in understanding how salinity affects floral induction and flowering, particularly how GA modulates these processes under ionic stress. Systems biology approaches could map the GA-ABA-ethylene signaling nexus under salinity, identifying key nodes for intervention. Furthermore, the relationship between GA and photosynthetic regulation under salinity remains underexplored. Studies must clarify how GA improves photosynthetic parameters (e.g., chlorophyll stability, Rubisco activity) and mitigates photoinhibition in chloroplasts. Research is also needed to dissect the role of co-regulated hormones (e.g., cytokinins, auxins) at intersection points that alter photosynthetic machinery under stress.

### 5.3. Optimizing GA_3_ Application Strategies with Agronomic Practices

While exogenous GA_3_ mitigates salinity stress, its efficacy depends on concentration, timing, and delivery methods. The optimal GA_3_ concentration varies across species. Lower doses often enhance growth, while higher doses may suppress it. Field trials are critical to validate lab-derived findings as soil heterogeneity, irrigation practices, and NaCl concentrations in water solutions significantly alter GA_3_ efficacy. Future studies should optimize foliar vs. seed-priming applications, particularly in field trials across diverse agroecologies. Nano-delivery systems for GA_3_, coupled with osmoprotectants like glycine betaine, could enhance uptake and reduce degradation. Combining GA_3_ with soil amendments (biochar, gypsum) or microbial inoculants (PGPR, mycorrhizae) could synergistically improve soil health and nutrient availability under salinity. For example, GA_3_-enhanced root architecture may facilitate deeper nutrient mining in saline soils, while microbial consortia could degrade phytotoxic ions. Drip irrigation systems delivering GA_3_ directly to root zones could minimize leaching and maximize resource efficiency.

### 5.4. Climate-Resilient Crop and Socio-Economic Development

The era of climate change exacerbates secondary salinization, breeding programs must prioritize traits like Na^+^ exclusion, stomatal density modulation, and chloroplast lipid stability. High-throughput phenotyping platforms could screen germplasm for GA_3_-responsive genotypes, linking traits to genomic markers. For instance, *Linum usitatissimum*’s proline-GA_3_ interplay under salinity could inspire analogous mechanisms in staple crops. The cost–benefit analysis of GA_3_ application at scale requires validation, particularly for smallholder farmers in arid regions. Long-term ecological studies must assess GA_3_ runoff impacts on non-target ecosystems. Participatory research involving farmers could co-develop GA_3_-based protocols tailored to local cultivars and saline hotspots, ensuring adoption and equity.

### 5.5. Technological Advancements in Stress Monitoring and Policy Frameworks

Remote sensing and AI-driven models could predict salinity stress in real time, enabling precision GA_3_ application. Hyperspectral imaging may detect early photosynthetic dysfunction (e.g., PSII efficiency declines), triggering automated GA_3_ treatments. Similarly, IoT-enabled soil sensors monitoring Na^+^/K^+^ ratios could inform dynamic GA_3_ dosing. Global initiatives, akin to the FAO’s Global Soil Partnership, should prioritize GA_3_ research in salinity management roadmaps. Policymakers must incentivize public–private partnerships for biostimulant development, while extension services educate farmers on GA_3_ best practices. Open-access databases cataloging GA_3_-responsive cultivars could accelerate climate adaptation. Despite progress in understanding salinity tolerance mechanisms, critical gaps persist, particularly in GA-mediated flowering regulation, photosynthetic recovery, and hormonal cross-talk. The integration of GA_3_ into holistic salinity management strategies holds transformative potential for sustainable agriculture. By bridging molecular insights with agronomic innovation, future research can unlock GA_3_’s full capacity to safeguard crop productivity in the rapidly salinizing world. However, success hinges on interdisciplinary collaboration, equitable technology transfer, and adaptive policies that prioritize resilience in vulnerable agroecosystems.

## Figures and Tables

**Table 1:** Interactive role of GA_3_ and salinity in a variety of crops.

Crops	Salinity Level	GA_3_ Dose (ppm)	Response (GA_3_ Effect vs. SALT-Only)	Source
Maize (*Zea mays* L.)	EC ≈ 7.8 dS m^−1^ (saline–calcareous soil)	100	GA_3_ (100 ppm) ↑ grain yield (+33%), ↑ chlorophyll (+27%), ↑ proline (+459%), ↑ K^+^/Na^+^ ratio, ↓ Na^+^ uptake (–25%)	[16]
Stevis (*Stevia rebaudiana* Bertoni)	80 mM	100	↑ aerial biomass (+54%), ↑ root biomass (+31%), ↑ leaf chlorophyll (+40%), ↓ MDA (–41%), ↓ H_2_O_2_ (–34%), ↑ SOD (+31%), ↑ CAT (+11%), ↑ PPO (+24%)	[45]
Pea (*Pisum sativum* L.)	EC = 8 dS m^−1^	100	↑ plant height (+27%), ↑ chlorophyll (+18%), ↑ RWC (+15%), ↑ K^+^ uptake (+22%), ↓ Na^+^ accumulation (–19%)	[44]
Rice (*Oryza sativa* L.)	100 mM	50	GA_3_ mitigated DELLA accumulation, restored PSII efficiency (Fv/Fm ↑ 19%), ↑ Rubisco activity (+22%), improved chlorophyll retention	[62]
Wheat (*Triticum aestivum* L.)	EC = 5.11 dS m^−1^	10	↑ plant water content (54.94 → 60.77%), ↑ shoot fresh weight (34.47 → 43.18 g), ↑ chlorophyll and RWC	[49]
Turnip (*Brassica rapa* L. subsp. rapa)	100 mM	100	↑ chlorophyll (+32%), ↑ SOD (+25%), ↑ CAT (+20%), ↓ H_2_O_2_ (–30%), ↑ photosynthetic rate (+18%)	[50]

## Data Availability

No new data were created or analyzed in this study.

## References

[1] Phour M., Sindhu S.S., Parihar M., Rakshit A., Adholeya A., Chen Y. (2024). Arbuscular mycorrhizal fungi: An eco-friendly technology for alleviation of salinity stress and nutrient acquisition in sustainable agriculture. Arbuscular Mycorrhizal Fungi in Sustainable Agriculture: Nutrient and Crop Management.

[2] Khalid M.F., Jawaid M.Z., Nawaz M., Shakoor R.A., Ahmed T. (2024). Employing titanium dioxide nanoparticles as biostimulant against salinity: Improving antioxidative defense and reactive oxygen species balancing in eggplant seedlings. Antioxidants.

[3] Boussora F., Triki T., Bennani L., Bagues M., Ben Ali S., Ferchichi A., Guasmi F. (2024). Mineral accumulation, relative water content and gas exchange are the main physiological regulating mechanisms to cope with salt stress in barley. Sci. Rep..

[4] Waheed R., Xie S., Rejman K. (2025). Inspect the influence of socio-economic, land, water, physical capital, and ecosystem on land degradation: In View of COP28. Land Degradation & Development.

[5] El-Ramady H., Prokisch J., Mansour H., Bayoumi Y.A., Shalaby T.A., Veres S., Brevik E.C. (2024). Review of crop response to soil salinity stress: Possible approaches from leaching to nano-management. Soil Syst..

[6] Hiteshkumar V., Kandoliya U.K., Singh P.P., Parakhiya M.V., Gore V. (2025). Effect of exogenous application of gibberellic acid and salicylic acid on antioxidative enzymes in Indian mustard irrigated with saline water. Plant Arch..

[7] United Nations, Department of Economic and Social Affairs, Population Division (2023). World Population Prospects 2023: Summary of Results.

[8] Nadeem M., Shahbaz M., Ahmad F., Waraich E.A. (2025). Enhancing wheat resistance to salinity: The role of gibberellic acid and β-Carotene in morphological, yielding and ionic adaptations. J. Ecol. Eng..

[9] Sarwar R., Zhu K.M., Jiang T., Ding P., Gao Y., Tan X.L. (2023). DELLAs directed gibberellins responses orchestrate crop development: A brief review. Crop Sci..

[10] Liu J., Wu Y., Dong G., Zhu G., Zhou G. (2023). Progress of research on the physiology and molecular regulation of sorghum growth under salt stress by gibberellin. Int. J. Mol. Sci..

[11] Chen Q., Li G., Duan J., Tang Z., Song Z., Liu H., Shi X. (2025). Gibberellin-induced germination enhancement in Chinese sour jujube seeds under cold stress. Plant Stress.

[12] Yari Kamrani Y., Shomali A., Aliniaeifard S., Lastochkina O., Moosavi-Nezhad M., Hajinajaf N., Talar U. (2022). Regulatory role of circadian clocks on ABA production and signaling, stomatal responses, and water-use efficiency under water-deficit conditions. Cells.

[13] Joshi S., Nath J., Singh A.K., Pareek A., Joshi R. (2022). Ion transporters and their regulatory signal transduction mechanisms for salinity tolerance in plants. Physiol. Plant..

[14] Chhabra R. (2022). Irrigation and salinity control. Salt-Affected Soils and Marginal Waters: Global Perspectives and Sustainable Management.

[15] Kumar P., Sharma P.K. (2020). Soil salinity and food security in India. Front. Sustain. Food Syst..

[16] Shahzad R., Harlina P.W., Ewas M., Zhenyuan P., Nie X., Gallego P.P., Khan S.U., Nishawy E., Khan A.H., Jia H. (2021). Foliar applied 24-epibrassinolide alleviates salt stress in rice (*Oryza sativa* L.) by suppression of ABA levels and upregulation of secondary metabolites. J. Plant Interact..

[17] Mukhopadhyay R., Sarkar B., Jat H.S., Sharma P.C., Bolan N.S. (2021). Soil salinity under climate change: Challenges for sustainable agriculture and food security. J. Environ. Manag..

[18] Iqbal N., Khan N.A., Ferrante A., Trivellini A., Francini A., Khan M.I.R. (2017). Ethylene role in plant growth, development and senescence: Interaction with other phytohormones. Front. Plant Sci..

[19] Ibrahimova U., Zivcak M., Gasparovic K., Rastogi A., Allakhverdiev S.I., Yang X., Brestic M. (2021). Electron and proton transport in wheat exposed to salt stress: Is the increase of the thylakoid membrane proton conductivity responsible for decreasing the photosynthetic activity in sensitive genotypes?. Photosynth. Res..

[20] Yildirim E., Taylor A., Spittler T. (2006). Ameliorative effects of biological treatments on growth of squash plants under salt stress. Sci. Hortic..

[21] Mohanta T.K., Bashir T., Hashem A., Abd_Allah E.F., Khan A.L., Al-Harrasi A.S. (2018). Early Events in Plant Abiotic Stress Signaling: Interplay Between Calcium, Reactive Oxygen Species and Phytohormones. J. Plant Growth Regul..

[22] Singh P., Choudhary K.K., Chaudhary N., Gupta S., Sahu M., Tejaswini B., Sarkar S. (2022). Salt stress resilience in plants mediated through osmolyte accumulation and its crosstalk mechanism with phytohormones. Front. Plant Sci..

[23] Liu H., Wang W., Wu H., Gong X., Moriguchi T. (2015). Polyamines function in stress tolerance: From synthesis to regulation. Front. Plant Sci..

[24] Nidhi Iqbal N., Khan N.A. (2025). Polyamines interaction with gaseous signaling molecules for resilience against drought and heat stress in plants. Plants.

[25] Ziaf K., Amjad M., Pervez M.A., Iqbal Q., Rajwana I.A., Ayyub M. (2009). Evaluation of different growth and physiological traits as indices of salt tolerance in hot pepper (*Capsicum annuum* L.). Pak. J. Bot.

[26] Dutta S., Jharna D.E., Islam M.J., Pipil M.H. (2025). Morpho-biochemical changes of chili (*Capsicum annuum* L) genotypes under different salt stress conditions. J. Bangladesh Agric. Univ..

[27] Shahid M.A., Sarkhosh A., Khan N., Balal R.M., Ali S., Rossi L., Gómez C., Mattson N., Nasim W., Garcia-Sanchez F. (2020). Insights into the physiological and biochemical impacts of salt stress on plant growth and development. Agronomy.

[28] Abou Seeda M.A., Abou El-Nour A.A., El-Bassiouny M.S., Abdallah M.S., El-Monem A.A. (2022). Impacts of salinity stress on plants and their tolerance strategies: A review. Middle East J. Appl. Sci..

[29] Muhammad I., Shalmani A., Ali M., Yang Q.-H., Ahmad H., Li F.B. (2021). Mechanisms regulating the dynamics of photosynthesis under abiotic stresses. Front. Plant Sci..

[30] Navarro-Torre S., Garcia-Caparrós P., Nogales A., Abreu M.M., Santos E., Cortinhas A.L., Caperta A.D. (2023). Sustainable agricultural management of saline soils in arid and semi-arid Mediterranean regions through halophytes, microbial and soil-based technologies. Environ. Exp. Bot..

[31] Hussain Q., Asim M., Zhang R., Khan R., Farooq S., Wu J. (2021). Transcription factors interact with ABA through gene expression and signaling pathways to mitigate drought and salinity stress. Biomolecules.

[32] Sharma A., Kumar V., Shahzad B., Ramakrishnan M., Sidhu G.P.S., Bali A.S., Handa N., Kapoor D., Yadav P., Khanna K. (2020). Photosynthetic response of plants under different abiotic stresses: A review. J. Plant Growth Regul..

[33] Takahashi Y., Joo H., Pankasem N., Hsu P., Schroeder J.I. (2025). Stomatal CO_2_ sensing in plants: Control of gas exchange and interactions with environmental stimuli. Plant Cell Physiol..

[34] Falcioni R., de Oliveira C.A., Vedana N.G., Mendonça W.A., Gonçalves J.V.F., Haubert D.d.F.d.S., de Matos D.H.S., Reis A.S., Antunes W.C., Crusiol L.G.T. (2025). Progressive Water Deficit Impairs Soybean Growth, Alters Metabolic Profiles, and Decreases Photosynthetic Efficiency. Plants.

[35] Dos Santos T.B., Ribas A.F., de Souza S.G.H., Budzinski I.G.F., Domingues D.S. (2022). Physiological responses to drought, salinity, and heat stress in plants: A review. Stresses.

[36] Zuo G., Zhang R., Feng N., Zheng D. (2024). Photosynthetic responses to salt stress in two rice (*Oryza sativa* L.) varieties. Agronomy.

[37] ELSabagh A., Mbarki S., Hossain A., Iqbal M.A., Islam M.S., Raza A., Llanes A., Reginato M., Rahman A., Mahboob W. (2021). Potential role of plant growth regulators in administering crucial processes against abiotic stresses. Front. Agron..

[38] ELSabagh A., Islam M.S., Hossain A., Iqbal M.A., Mubeen M., Waleed M., Reginato M., Battaglia M., Ahmed S., Rehman A. (2022). Phytohormones as growth regulators during abiotic stress tolerance in plants. Front. Agron..

[39] Gao S., Chu C. (2020). Gibberellin metabolism and signaling: Targets for improving agronomic performance of crops. Plant Cell Physiol..

[40] Shah S.H., Islam S., Mohammad F., Siddiqui M.H. (2023). Gibberellic acid: A versatile regulator of plant growth, development and stress responses. J. Plant Growth Regul..

[41] Castro-Camba R., Sánchez C., Vidal N., Vielba J.M. (2022). Plant development and crop yield: The role of gibberellins. Plants.

[42] Islam S., Park K., Xia J., Kwon E., Kim D.Y. (2025). Structural insights of gibberellin-mediated DELLA protein degradation. Molecular Plant.

[43] KS A., Puthur J.T., Dhankher O.P. (2025). Plant Cell Wall Remodeling under Toxic Metal Stress: Structural Adaptation and Functional Implications. Environ. Sci. Technol..

[44] Gurmani A.R., Wang X., Rafique M., Jawad M., Khan A.R., Khan Q.U., Ahmed R., Fiaz S. (2022). Exogenous application of gibberellic acid and silicon to promote salinity tolerance in pea (*Pisum sativum* L.) through Na^+^ exclusion. Saudi J. Biol. Sci..

[45] Janah I., Ben-Laouane R., Elhasnaoui A., Anli M., Meddich A. (2024). The induction of salt stress tolerance by gibberellic acid treatment in Stevia rebaudiana Bertoni plants. Int. J. Plant Biol..

[46] Tuna A.L., Kaya C., Dikilitas M., Higgs D. (2008). The combined effects of gibberellic acid and salinity on some antioxidant enzyme activities, plant growth parameters and nutritional status in maize plants. Environ. Exp. Bot..

[47] Yadav N., Kumar A., Sawariya M., Kumar N., Mehra H., Kumar S., Kaur V., Arya S.S. (2024). Effect of GA3 and calcium on growth, biochemical, and fatty acid composition of linseed under chloride-dominated salinity. Environ. Sci. Pollut. Res. Int..

[48] Ullah A., Hazrat A., Khan B.A., Saqib S., Ullah F. (2025). Mitigating lead stress in barley using gibberellic acid (GA_3_): Effects on morpho-physiological and biochemical parameters. J. Plant Growth Regul..

[49] Anwar T., Munwwar F., Qureshi H., Siddiqi E.H., Hanif A., Anwaar S., Gul S., Waheed A., Alwahibi M.S., Kamal A. (2023). Synergistic effect of biochar-based compounds from vegetable wastes and gibberellic acid on wheat growth under salinity stress. Sci. Rep..

[50] Fatima A., Umbreen S., Sadia S., Waheed M., Arshad F., Malik M.R., Hashem A., Kumar A., Abd_Allah E.F. (2024). Mitigation of salinity-induced adverse effects through exogenous application of gibberellic acid in turnip (*Brassica rapa* L.). Cogent Food Agric..

[51] Chauhan A., AbuAmarah B.A., Kumar A., Verma J., Ghramh H.A., Khan K.A., Ansari M.J. (2019). Influence of gibberellic acid and different salt concentrations on germination percentage and physiological parameters of oat cultivars. Saudi J. Biol. Sci..

[52] Dhansu P., Kumar R., Kumar A., Vengavasi K., Raja A.K., Vasantha S., Meena M.R., Kulshreshtha N., Pandey S.K. (2022). Differential physiological traits, ion homeostasis and cane yield of sub-tropical sugarcane varieties in response to long-term salinity stress. Sustainability.

[53] Jhanji S., Goyal E., Chumber M., Kaur G. (2024). Exploring fine tuning between phytohormones and ROS signaling cascade in regulation of seed dormancy, germination and seedling development. Plant Physiol. Biochem..

[54] Xiong H., He H., Chang Y., Miao B., Liu Z., Wang Q., Xiong L. (2025). Multiple roles of NAC transcription factors in plant development and stress responses. J. Integr. Plant Biol..

[55] Hedden P. (2020). The current status of research on gibberellin biosynthesis. Plant Cell Physiol..

[56] Yu B., Hu Y., Hou X. (2025). More than flowering: CONSTANS plays multifaceted roles in plant development and stress responses. J. Integr. Plant Biol..

[57] Zhou J., Li Z., Xiao G., Zhai M., Pan X., Huang R., Zhang H. (2020). CYP71D8L is a key regulator involved in growth and stress responses by mediating gibberellin homeostasis in rice. J. Exp. Bot..

[58] Attia H., Alamer K., Algethami B., Zorrig W., Hessini K., Gupta K., Gupta B. (2022). Gibberellic acid interacts with salt stress on germination, growth and polyamine gene expression in fennel (*Foeniculum vulgare Mill*.) seedlings. Physiol. Mol. Biol. Plants.

[59] Xu S., Huang Y., Zhang R., Niu L., Long H. (2024). Appropriate nitrogen application for alleviation of soil moisture-driven growth inhibition of okra (*Abelmoschus esculentus* L. (*Moench*)). Horticulturae.

[60] Das S., Shil S., Rime J., Alice A.K., Yumkhaibam T., Mounika V., Singh A.P., Kundu M., Lalhmangaihzuali H.P., Hazarika T.P. (2025). Phytohormonal signaling in plant resilience: Advances and strategies for enhancing abiotic stress tolerance. Plant Growth Regul..

[61] Kubalová M., Griffiths J., Müller K., Levenets L., Tylová E., Tarkowská D., Jones A.M., Fendrych M. (2025). Gibberellin-deactivating GA2OX enzymes act as a hub for auxin-gibberellin cross talk in Arabidopsis thaliana root growth regulation. Proc. Natl. Acad. Sci. USA.

[62] Li Y., Cheng Y., Wei F., Liu Y., Zhu R., Zhao P., Shang Z. (2024). Arabidopsis thaliana MYC_2_ and MYC_3_ are involved in ethylene-regulated hypocotyl growth as negative regulators. Int. J. Mol. Sci..

[63] Colebrook E.H., Thomas S.G., Phillips A.L., Hedden P. (2014). The role of gibberellin signalling in plant responses to abiotic stress. J. Exp. Biol..

[64] Basit F., Alyafei M., Hayat F., Al-Zayadneh W., El-Keblawy A., Sulieman S., Sheteiwy M.S. (2025). Deciphering the role of glycine betaine in enhancing plant performance and defense mechanisms against environmental stresses. Front. Plant Sci..

[65] Trugman A.T., Anderegg L.D. (2025). Source vs. sink limitations on tree growth: From physiological mechanisms to evolutionary constraints and terrestrial carbon cycle implications. New Phytol..

[66] Iqbal M., Zahoor M., Akbar M., Ahmad K., Hussain S., Munir S., Ali M., Arshad N., Masood H., Zafar S. (2022). Alleviating the deleterious effects of salt stress on wheat (*Triticum aestivum* L.) By foliar application of gibberellic acid and salicylic acid. Appl. Ecol. Environ. Res..

[67] Manasa H.M., Doddagoudar S.R., Gowda B., Shakuntala N.M., Mahantashivayogayya K., Lakshmikanth (2024). Influence of seed priming and foliar spray on seed germination, seedling growth, total carbohydrate and protein content of resultant rice (*Oryza Sativa* L.) seeds under salinity. Seed Res..

[68] Iqbal M., Ashraf M. (2013). Gibberellic acid mediated induction of salt tolerance in wheat plants: Growth, ionic partitioning, photosynthesis, yield and hormonal homeostasis. Environ. Exp. Bot..

[69] Mukarram M., Mohammad F., Naeem M., Khan M.M.A., Naeem M., Aftab T., Khan M.M.A. (2021). Exogenous Gibberellic Acid Supplementation Renders Growth and Yield Protection Against Salinity Induced Oxidative Damage Through Upregulating Antioxidant Metabolism in Fenugreek (*Trigonella foenum-graceum* L.). Fenugreek.

[70] Hasanuzzaman M., Bhuyan M.B., Zulfiqar F., Raza A., Mohsin S.M., Al Mahmud J., Fujita M., Fotopoulos V. (2020). Reactive Oxygen Species and Antioxidant Defense in Plants under Abiotic Stress: Revisiting the Crucial Role of a Universal Defense Regulator. Antioxidants.

[71] Rastegar S., Sayyad-Amin P. (2025). GABA and Oxidative Stress and the Regulation of Antioxidants. GABA in Plants: Biosynthesis, Plant Development, and Food Security.

[72] Ikram M., Khalid B., Batool M., Ullah M., Zitong J., Rauf A., Rao M.J., Rehman H.U., Kuai J., Xu Z. (2025). Secondary metabolites as biostimulants in salt stressed plants: Mechanisms of oxidative defense and signal transduction. Plant Stress.

[73] Hameed A., Ahmed M.Z., Hussain T., Aziz I., Ahmad N., Gul B., Nielsen B.L. (2021). Effects of Salinity Stress on Chloroplast Structure and Function. Cells.

[74] Zuo G. (2025). Non-photochemical quenching (NPQ) in photoprotection: Insights into NPQ levels required to avoid photoinactivation and photoinhibition. New Phytol..

[75] Zahra N., Al Hinai M.S., Hafeez M.B., Rehman A., Wahid A., Siddique K.H., Farooq M. (2022). Regulation of photosynthesis under salt stress and associated tolerance mechanisms. Plant Physiol. Biochem..

[76] Atta K., Mondal S., Gorai S., Singh A.P., Kumari A., Ghosh T., Roy A., Hembram S., Gaikwad D.J., Mondal S. (2023). Impacts of salinity stress on crop plants: Improving salt tolerance through genetic and molecular dissection. Front. Plant Sci..

[77] Gong L., Hazzazi Y., Alfaifi T., Alabdallah N.M., Alnusaire T.S., Altihani F.A., Dhawi F., Alharbi B.M., Hasan M. (2025). Root-to-shoot hormonal and hydraulic signals in stomatal regulation during drought. J. Plant Growth Regul..

[78] Ding F., Fan X., Tian R., Wang M., Sun Z. (2025). Crosstalk of Abscisic Acid with Other Hormones and Signaling Molecules in Tomato Cold Stress Tolerance. Horticulturae.

[79] Asif Mahamud M., Imran S., Paul N.C., Rabbi R.H.M., Jahan N., Sarker P., Hoque M.N., Sumi M.J., Asaduzzaman M., Rehman S.U. (2025). An overview and current progress of gibberellic acid-mediated abiotic stress alleviation in plants. Plant Soil Environ..

[80] Bharath P., Gahir S., Raghavendra A.S. (2021). Abscisic Acid-Induced Stomatal Closure: An Important Component of Plant Defense Against Abiotic and Biotic Stress. Front. Plant Sci..

[81] Upreti K.K., Bhatt R.M., Panneerselvam P., Varalakshmi L.R. (2016). Morpho-Physiological Responses of Grape Rootstock ‘Dogridge’ to Arbuscular Mycorrhizal Fungi Inoculation Under Salinity Stress. Int. J. Fruit Sci..

[82] Sakakibara H. (2021). Cytokinin biosynthesis and transport for systemic nitrogen signaling. Plant J..

[83] Hossain A., Pamanick B., Venugopalan V.K., Ibrahimova U., Rahman M.A., Siyal A.L., Maitra S., Chatterjee S., Aftab T. (2022). Emerging roles of plant growth regulators for plants adaptation to abiotic stress–induced oxidative stress. Emerging Plant Growth Regulators in Agriculture.

[84] Mbarki S., Sytar O., Cerda A., Zivcak M., Rastogi A., He X., Brestic M. (2018). Strategies to mitigate the salt stress effects on photosynthetic apparatus and productivity of crop plants. Salinity Responses and Tolerance in Plants, Volume 1: Targeting Sensory, Transport and Signaling Mechanisms.

[85] Lupo Y., Prashanth K., Lazarovitch N., Fait A., Rachmilevitch S. (2023). Importance of leaf age in grapevines (*Vitis* spp.) under salt stress. Sci. Hortic..

[86] Jia S., Zhou Q., Yuan S., Wang Y., Zhang Z. (2025). Molecular and Physiological Mechanisms Underlying Submerged Germination in Rice. Biology.

[87] Kosakivska I., Babenko L., Voytenko L., Vasyuk V., Shcherbatiuk M., Romanenko K. (2025). Natural growth regulators in enhancing cereal crop resistance to lead contamination. Cereal Research Communications.

[88] Danish S., Sana S., Hussain M.B., Dawar K., Almoallim H.S., Ansari M.J., Hareem M., Datta R. (2024). Effect of methyl jasmonate and GA3 on canola (*Brassica napus* L.) growth, antioxidants activity, and nutrient concentration cultivated in salt-affected soils. BMC Plant Biol..

[89] Khan S., Alvi A.F., Khan N.A. (2024). Role of ethylene in the regulation of plant developmental processes. Stresses.

[90] Abbas H.M.K., Askri S.M.H., Ali S., Fatima A., Qamar M.T.U., Xue S.D., Muhammad Z., Akram W., Zhong Y.J. (2022). Mechanism associated with brassinosteroids crosstalk with gibberellic acid in plants. Brassinosteroids Signalling: Intervention with Phytohormones and Their Relationship in Plant Adaptation to Abiotic Stresses.

[91] Khan N. (2025). Decoding the Long-Term Impacts of Genetic Modifications in Hormone Pathways on Plant Physiology and Ecosystem Stability. Environ. Rev..

[92] Mu Y., Cao K., Lu J., Wang J., Li X., Zhang X. (2025). Molecular Mechanisms of Seed Dormancy Release in Paeonia lactiflora Revealed through Transcriptomic and Metabolomic Analysis. https://www.researchsquare.com/article/rs-7250070/v1.

[93] Wang X., Zong N., Wang X., Niu J., Zhang X., Shu K., Hui W. (2025). Gamma-aminobutyric acid (GABA) releases seed dormancy by orchestrating abscisic acid and gibberellin metabolism and signaling. BMC Plant Biol..

[94] Wang X., Wen H., Suprun A., Zhu H. (2025). Ethylene signaling in regulating plant growth, development, and stress responses. Plants.

[95] Panozzo A., Bolla P.K., Barion G., Botton A., Vamerali T. (2025). Phytohormonal Regulation of Abiotic Stress Tolerance, Leaf Senescence and Yield Response in Field Crops: A Comprehensive Review. BioTech.

[96] Voisin A., Reidy B., Parent B., Rolland G., Redondo E., Gerentes D., Tardieu F., Muller B. (2006). Are ABA, ethylene or their interaction involved in the response of leaf growth to soil water deficit? An analysis using naturally occurring variation or genetic transformation of ABA production in maize. Plant Cell Environ..

[97] Mishra A., Marwal A., Tailor S., Jain K., Malik A., Suthar M., Meena M. (2024). Overview of cell signaling response under plant stress. Molecular Dynamics of Plant Stress and Its Management.

[98] Xie Z., Jin L., Sun Y., Zhan C., Tang S., Qin T., Huang J. (2024). OsNAC120 balances plant growth and drought tolerance by integrating GA and ABA signaling in rice. Plant Commun..

[99] Sakouhi L., Hussaan M., Murata Y., Chaoui A. (2024). Role of calcium signaling in cadmium stress mitigation by indol-3-acetic acid and gibberellin in chickpea seedlings. Environ. Sci. Pollut. Res..

[100] Ghosh S., Bheri M., Pandey G.K. (2021). Delineating calcium signaling machinery in plants: Tapping the potential through functional genomics. Curr. Genom..

[101] Ikram M., Batool M., Ullah M., Khalid B., El-Badri A.M., Mohamed I.A.A., Zhang L., Kuai J., Xu Z., Zhao J. (2025). Molecular alchemy: Converting stress into resilience via secondary metabolites and calcium signaling in rice. Rice.

[102] Li W., Yong Y., Zhang Y., Lyu Y. (2019). Transcriptional regulatory network of GA floral induction pathway in LA hybrid lily. Int. J. Mol. Sci..

